# Measuring XNA polymerase fidelity in a hydrogel particle format

**DOI:** 10.1093/nar/gkaf038

**Published:** 2025-01-29

**Authors:** Esau L Medina, John C Chaput

**Affiliations:** Department of Pharmaceutical Sciences, University of California, Irvine, CA 92697-3958, United States; Department of Pharmaceutical Sciences, University of California, Irvine, CA 92697-3958, United States; Department of Chemistry, University of California, Irvine, CA 92697-3958, United States; Department of Molecular Biology and Biochemistry, University of California, Irvine, CA 92697-3958, United States

## Abstract

Growth in the development of engineered polymerases for synthetic biology has led to renewed interest in assays that can measure the fidelity of polymerases that are capable of synthesizing artificial genetic polymers (XNAs). Conventional approaches require purifying the XNA intermediate of a replication cycle (DNA → XNA → DNA) by denaturing polyacrylamide gel electrophoresis, which is a slow, costly, and inefficient process that requires a large-scale transcription reaction and careful extraction of the XNA strand from the gel slice. In an effort to streamline the assay, we developed a purification-free approach in which the XNA transcription and reverse transcription steps occur inside the matrix of a hydrogel-coated magnetic particle. Accordingly, a DNA primer cross-linked throughout the gel matrix is annealed to a template of defined sequence and extended with XNA. Following removal of the DNA template, the XNA product strand is copied back into DNA, recovered, amplified, cloned, and sequenced. Performing the replication cycle in the hydrogel format drastically reduces the time and reaction scales required to measure the fidelity of an XNA polymerase, making it easier to evaluate the properties of a range of candidate XNA polymerases.

## Graphical Abstract

**Graphical Abstract:**
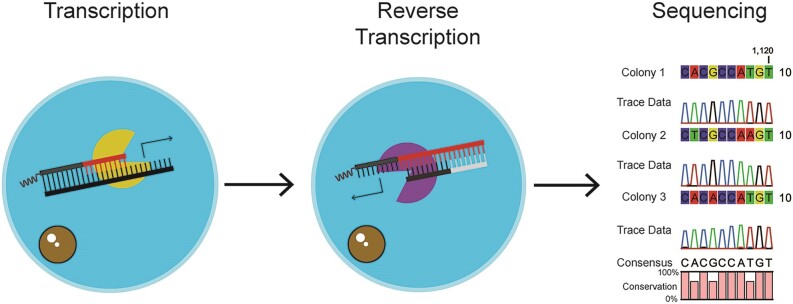

## Introduction

DNA polymerases are the cornerstone of biotechnology, responsible for accurately synthesizing DNA in a myriad of applications ranging from anthropology to personalized diagnostics [1]. Natural DNA polymerases tend to function with error rates in the range of one mistake per 10^5^–10^6^ nucleotide (nt) incorporations [2]. This level of accuracy can be improved with proofreading activity in which the 3′–5′ exonuclease domain of a polymerase, if present, is able to identify and remove nucleotides that have been incorrectly added to 3′ end of the extended strand [3]. Because DNA synthesis has become an indispensable component of many biotechnology applications, several methods have been developed to measure the fidelity of DNA synthesis. In one such case, Tindall and Kunkel showed that the *lacZα* gene in M13 bacteriophage could be used to measure polymerase error rates in *Escherichia coli* using a white to blue color change in colonies grown on solid agar media [4]. Subsequently, Goodman *et al.* established a cell-free assay that calculates fidelity by measuring the insertion kinetics of single nucleotide addition reactions for matched and mismatched primer extension reactions [5, 6]. Although this assay is still used to evaluate the rates of nucleotide synthesis, classic approaches for measuring fidelity have largely been replaced by DNA sequencing strategies that make it easier to identify and quantify mutations, including insertions and deletions (INDELs) [7].

Polymerase fidelity measurements are currently experiencing renewed interest due to growth in the development of engineered polymerases for applications in synthetic biology [8]. Polymerases have now been produced that can synthesize artificial genetic polymers (XNAs) with a range of backbone chemistries, including 2′-fluoroarabino nucleic acid (FANA) [9], 1,5-anhydrohexitol nucleic acid (HNA) [9], threose nucleic acid (TNA) [10], locked nucleic acid [11], phosphonomethylthreosyl nucleic acid [12], phosphoramidite DNA [13], and various 2′-modified RNA analogs [14–16]. These unnatural genetic polymers have gained attention due to their unique physicochemical properties, which may include increased stability against nuclease digestion [17], enhanced thermodynamics of Watson–Crick base pairing [18–20], and resistance to acid-mediated degradation [21, 22]. Polymerase fidelity is typically determined by sequencing the product of a complete cycle of XNA replication (DNA → XNA → DNA), which involves a series of primer extension reactions whereby a DNA template of defined sequence is transcribed into XNA, purified, reverse transcribed back into complementary DNA (cDNA), amplified using the polymerase chain reaction (PCR), cloned, and sequenced. However, performing this cycle in bulk solution is a costly and tedious procedure, as transcription reactions must be performed on large scales (typically 1 ml) and physical separation of the resulting XNA product from the DNA template requires denaturing polyacrylamide gel electrophoresis (PAGE) and electroelution. Additionally, separation of the XNA strand within the gel can be difficult for XNA systems with high affinity to their cDNA [23].

Motivated by a desire to accelerate the replication cycle of an XNA polymerase fidelity assay, we sought to develop a method that avoided the time-consuming process of PAGE purification and allowed the molecular biology steps to be performed on smaller scales. This latter concern is often overlooked but important to the field, as many XNA triphosphates (xNTPs) can only be obtained by chemical synthesis, which makes them valuable reagents for synthetic biology. Our approach to this problem involves performing the replication cycle in a hydrogel particle display format in which the initial DNA primer is covalently distributed throughout the gel matrix of a polyacrylamide-encapsulated magnetic particle. The hydrogel provides a favorable solution-like environment that allows reagents and enzymes to flow in and out of the gel matrix, making it easy to transcribe and reverse transcribe the XNA strand without the need for separate purification steps, as templates and reagents can be removed by simply washing the particles with an appropriate buffer. In this format, the entire assay can be completed in as little as 1–2 days, as compared to 1 week for the traditional solution-based approach, and miniaturization reduces the consumption of xNTP by 10-fold. We suggest that the benefits of increased speed and reduced consumables make hydrogel particles an ideal format for assessing XNA polymerase fidelity.

## Materials and methods

### Chemicals and reagents

DNA oligonucleotides, including the 5′-modified acrydite primer, were purchased from Integrated DNA Technologies (Coralville, IA) with standard desalting purification (Supplementary Table S1). TNA triphosphates were obtained by chemical synthesis as described previously [24, 25]. RNA and FANA triphosphates were purchased from TriLink Biotechnologies (San Diego, CA). HNA triphosphates were generously provided as a gift from Piet Herdewijn. ThermoPol (10×) buffer was purchased from New England Biolabs (Ipswich, MA). TOPO-TA cloning kit, ethylenediaminetetraacetic acid (EDTA), Tris, Dynabeads M-270 carboxylic acid 2.8 μM, isopropyl alcohol, ethanol, and *N*,*N*,*N*′,*N*′-tetramethylethane-1,2-diamine (TEMED) were purchased from Thermo Fisher Scientific (Waltham, MA). Silicone emulsifiers KF-6012, KF-6038, and DM-FLUID-A-6cs were purchased from Shin-Etsu Chemical Co. (Chiyoda City, Tokyo, Japan). Ammonium persulfate, mineral oil, and Triton X-100 were purchased from Sigma–Aldrich (St Louis, MO). Acrylamide (40%) and bisacrylamide (19:1) and sodium dodecyl sulfate (SDS) were purchased from Bio-Rad (Hercules, CA). Plasmid mini-prep kit was purchased from Biomiga (San Diego, CA).

### Expression and purification of recombinant DNA/XNA polymerases

The expression and purification of thermal stable recombinant XNA polymerases were performed as previously described [26]. In brief, DH5α competent *E. coli* cells were transformed with a pGDR11 vector encoding the polymerase and grown and expressed in liquid LB media supplemented with carbenicillin. Cells were centrifuged, resuspended in lysis/equilibration buffer (50 mM NaCl, 10 mM Tris–HCl, pH 7.5, 10% glycerol), sonicated, heat shocked (70°C, 1 h), precipitated on ice (>30 min), and centrifuged to pellet the cellular debris. Supernatant was collected and treated with 10% (v/v) polyethylenimine (PEI) for 15 min. Lysate was centrifuged to remove nucleic acids precipitated by the PEI. The protein was then precipitated overnight with ammonium sulfate. After incubation, the protein pellet was resuspended in lysis/equilibration buffer. Polymerases were purified on a heparin affinity column using a concentration gradient of NaCl starting at 250 mM to 1000 mM (10 mM Tris–HCl, pH 7.5, 10% glycerol). All polymerases were buffer exchanged into 500 mM NaCl storage buffer (10 mM Tris–HCl, pH 7.5, 10% glycerol) and concentrated using a 3K Amicon Ultra-0.5 spin column.

### Formation of DNA primer-functionalized hydrogel particles

The hydrogel particles were prepared as previously described [14, 15]. A 40% acrylamide/bisacrylamide (19:1) mixture was diluted to a 6% stock with nuclease-free Milli-Q water and purged under argon with a blunt needle (10 min). The oil mixture [KF-6038:mineral oil:DM-FLUID-A-6cs; 4%:20%:76% (w/w/w)] was combined at 24°C (60 min) and purged with argon (10 min). An aliquot of M-270 carboxylic acid Dynabeads (∼2 × 10^8^ beads) was transferred to a 1.5 ml centrifuge tube, collected on a magnetic stand, and the storage buffer was removed. The Dynabeads were resuspended in 6% acrylamide/bisacrylamide (264 μl), acrydite PBS8 primer (30 μl, 100 μM), and 0.6% ammonium persulfate (6 μl) in a total volume of 300 μl and pipette mixed. A layer of the oil mixture (900 μl) was added to the top of the resuspended Dynabeads (1200 μl total volume). The oil layer was degassed with argon via a 23-gauge needle (10 min). An aliquot of TEMED (1 μl) was added to the oil layer and the space above the oil layer was purged with argon gas (1 min). The oil and aqueous layers were mixed by placing the centrifuge tube into a BeadBug 6 (Benchmark) at 2500 rpm for 65 s, and then incubated on ice for 2 h. After the incubation, the centrifuge tube was placed on a magnetic stand, hydrogel particles were pulled down, and the supernatant was removed. The hydrogel particles were washed with 1 ml of breaking buffer [10 mM Tris–HCl, pH 7.5, 100 mM NaCl, 1 mM EDTA, 1% (v/v) SDS, and 1% (v/v) Triton X-100]. Additional washes were performed until all visible oil was removed from the tube. The hydrogels were resuspended in 1 ml of breaking buffer and agitated (500 rpm) overnight at 24°C. Two additional washes were performed before resuspending the hydrogels in 1 ml of breaking buffer. An aliquot (1 μl) of the resuspended hydrogels was diluted in breaking buffer (39 μl) and counted on a hemocytometer to determine the concentration of the stock.

### XNA synthesis on hydrogels

An aliquot of hydrogel particles (∼12 million hydrogels) was placed into a 1.5 ml centrifuge tube, pulled down using a magnetic stand, and the storage buffer was removed. The hydrogels were resuspended and washed twice with 1× ThermoPol buffer (200 μl). In a total volume of 100 μl for each XNA transcription reaction, hydrogels were resuspended in nuclease-free Milli-Q water, 1× ThermoPol buffer (10 μl, 10×), single-stranded DNA template (2 μl, 100 μM), and 1% KF-6012 (1 μl) and heated at 95°C for 3 min. The reaction mixture was snap-cooled on ice for 5 min to promote primer/template annealing. To initiate XNA synthesis, 2 μl of xNTPs (5 mM stock) and 20 μl of XNA polymerase (10 μM stock) were added, mixed, and incubated at 55°C on a ThermoMixer C for 2–4 h, depending on the XNA type. Afterward, the supernatant was removed, and the hydrogels were washed twice with 100 μl of 100 mM NaOH containing 0.05% Tween 20 with a 3 min incubation step to help denature the template and neutralized with an equivalent amount of breaking buffer. Hydrogels were stored at 4°C post-template stripping.

### XNA reverse transcription on hydrogels and cDNA purification

The hydrogel particles displaying the transcribed XNA sequences were washed twice with 1× ThermoPol buffer (100 μl). In a total reaction volume of 100 μl, the hydrogels were resuspended in nuclease-free Milli-Q water, 1× ThermoPol buffer (10 μl, 10×), PBS7/PBS9 overhang primer (2 μl, 100 μM) containing a two-nucleotide mismatch (TT–TT) in the primer binding region, 0.01% KF-6012 (1 μl, 1%), and 3 mM magnesium sulfate (3 μl, 100 mM) and heated at 95°C for 3 min. The reaction mixture was snap-cooled on ice for 5 min to promote primer/template annealing. To initiate the reaction, 10 μl of 2′-deoxynucleoside triphosphates (dNTPs; 5 mM stock) and 20 μl of Bst-LF (10 μM stock) were added, mixed, and the reaction was incubated at 50°C in a ThermoMixer C for 4–8 h, depending on the XNA.

To collect and purify the cDNA, the hydrogels were collected using a magnetic stand, the supernatant was removed, and 100 μl of 100 mM NaOH containing 0.05% Tween 20 was added. Following a brief incubation (24°C, 3 min), the hydrogels were collected (1 min), and the supernatant was removed and neutralized with an equivalent volume of 3 M NaOAc (pH 6–7). The cDNA was precipitated with cold 100% isopropyl alcohol (3 equiv.) and incubated on dry ice (>15 min). Precipitated cDNA was centrifuged for 1 h at 4°C (13 200 rpm), washed with cold 70% ethanol and centrifuged for 20 min at 4°C (13 200 rpm) twice, and air-dried for 30 min at 24°C. The pellet was resuspended with 10 μl nuclease-free water and the concentration was measured by NanoDrop.

### Fidelity assay

The cDNA was PCR amplified with Taq DNA polymerase, using the PBS8/PBS9 primer pair. Typical thermocycling conditions were as follows: denature at 95°C for 2 min, followed by 30 cycles of denaturing at 95°C for 2 min, annealing at 58°C for 15 s, extension at 72°C for 2.5 min, and a final polishing step 72°C for 1 min. The amplicons were ligated into a TOPO vector using a TOPO-TA kit and cloned into DH5α competent *E. coli* cells. Individual clones were picked, grown in liquid LB media, and sequenced (Primordium Labs, Arcadia, CA). DNA sequences were aligned and analyzed with the initial DNA template using MEGA11 and CLC Main. Error rates were determined as ${\mu}_{{\rm exp} \to {\rm obs}} = ( {{{\# {\rm observed}}}/{{\# {\rm expected}}}}) \times 1000.$ The total error rate was determined by summing the error rate for each substitution.

## Results and discussion

Recent advances have made it possible to generate hydrogel particles, magnetic beads coated with a polyacrylamide matrix, in a microfluidics-free reaction format [27]. Particles produced in this way are remarkably uniform in size (∼5 Å diameter), stable to elevated temperatures, and amenable to storage for months at 4°C. The porous nature of the gel matrix makes it possible to perform molecular biology reactions on DNA strands that have been cross-linked into the gel matrix using a 5′ acrydite functional group that is now commonly available as a standard modification from custom oligonucleotide synthesis companies. Examples demonstrated to date include the transcription and translation of genes, template-directed XNA synthesis, and ELISA-based screening of XNA aptamers [27–29]. In these systems, an NaOH stripping buffer is used to remove the noncovalently bound strand, which is an efficient process, even for XNAs with enhanced affinity for DNA. Full-length XNA products generated in a polymerase-mediated primer extension reaction are readily detected by fluorescence *in situ* hybridization (FISH), demonstrating compatibility with multiple conventional technologies, including flow cytometry [27].

Given the simplicity of their assembly and ease of use, we hypothesized that hydrogel particles offered a convenient method for accelerating the replication cycle required to measure the fidelity of an XNA polymerase. We envisioned an experimental format in which hydrogel particles would be prepared with a defined DNA primer that was covalently cross-linked throughout the gel matrix (Supplementary Fig. S1A). For XNA transcription, a DNA template would be annealed to the primer and extended with XNA using an XNA polymerase and the cognate xNTPs. Following transcription, the DNA template would be removed by washing the particles with a high-pH buffer (Supplementary Fig. S1B) to denature the heteroduplex. The covalently bound XNA product would then serve as the template for reverse transcription (RT). Synthesis of the cDNA strand using a suitable polymerase and dNTPs completes the replication cycle. Finally, the cDNA strand is recovered, amplified, cloned, and sequenced to determine the aggregate fidelity of XNA replication (Supplementary Fig. S1C).

**Figure 1.**
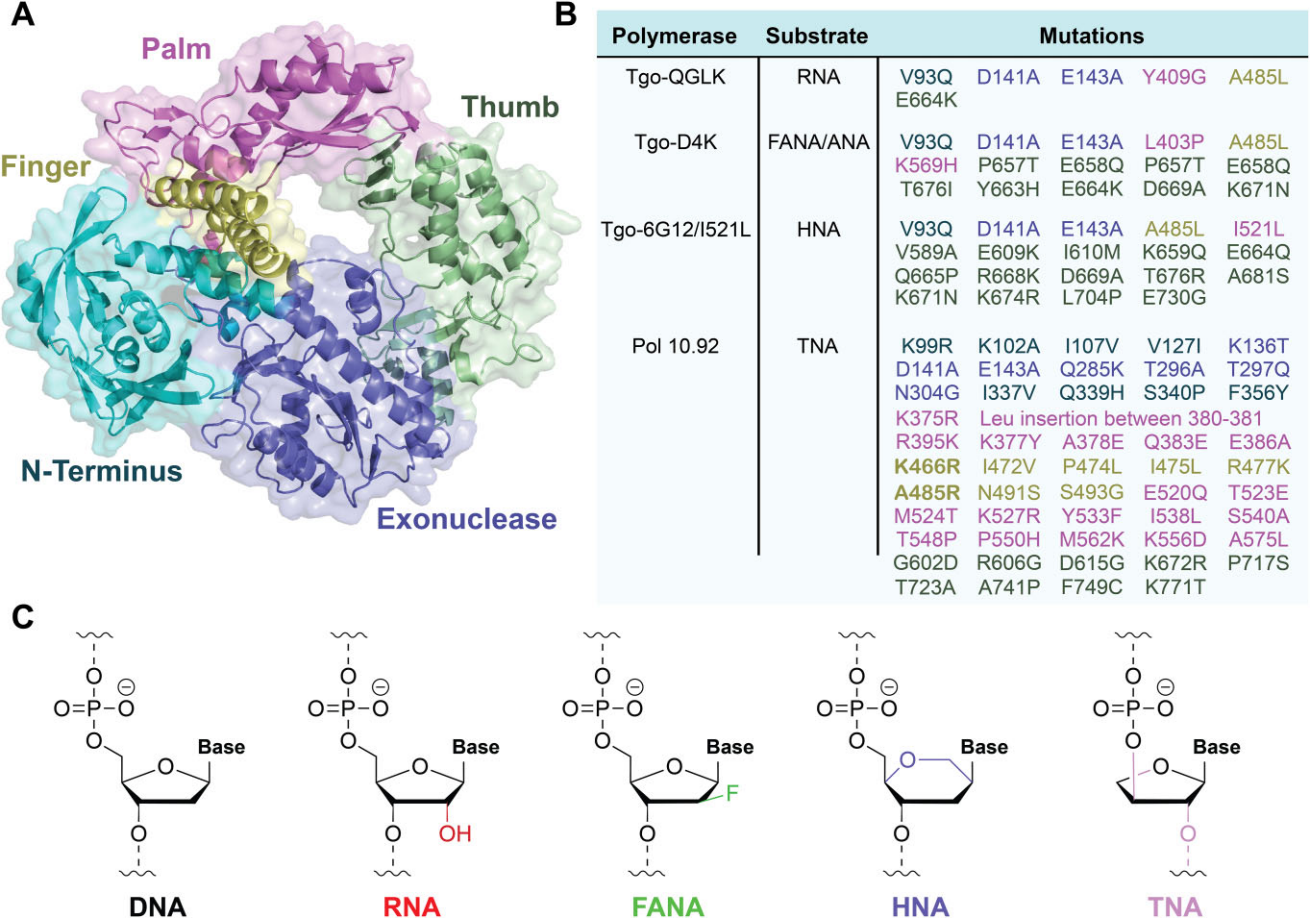
Engineered B-family XNA polymerases. (**A**) Crystal structure of a B-family DNA polymerase isolated from *Thermococcus gorgonarius* (Tgo), highlighting the N-terminal domain, exonuclease domain, and the palm, finger, and thumb subdomains. (**B**) Amino acid mutations elucidated by directed evolution experiments that confer RNA, FANA, ANA, HNA, and TNA synthesis activity. Mutations are color-coded to their respective domain/subdomain location. (**C**) Chemical structures of each XNA system, showing modifications to the sugar moiety.

We began by focusing on hyperthermophilic archaeal B-family polymerases that have been engineered in the laboratory to synthesize XNA (Fig. 1A) [30]. This ancient family of archaeal DNA polymerases has been used as the starting point for engineering numerous XNA polymerases. It is hypothesized that these hyperthermophilic polymerases found in deep ocean vents and hot springs are highly tolerant to mutations due to their structural stability, which can withstand extremely elevated temperatures for long durations of time [31]. Although many XNA polymerases have been developed to date, the current study focused on those that were engineered to synthesize RNA (Tgo-QGLK) [32], FANA (Tgo-D4K) [9], HNA (Tgo-6G12/I521L) [9], and TNA (pol 10–92) [33]. The mutations associated with these polymerases and the chemical structures of the XNA backbones are provided in Fig. 1. These XNA systems have been studied in molecular evolution experiments designed to explore the functional properties of alternative genetic polymers of life [34, 35], as well as investigate the potential for synthetic genetic polymers to contribute to areas of synthetic biology and molecular medicine [9, 23, 29, 36–38].

**Figure 2.**
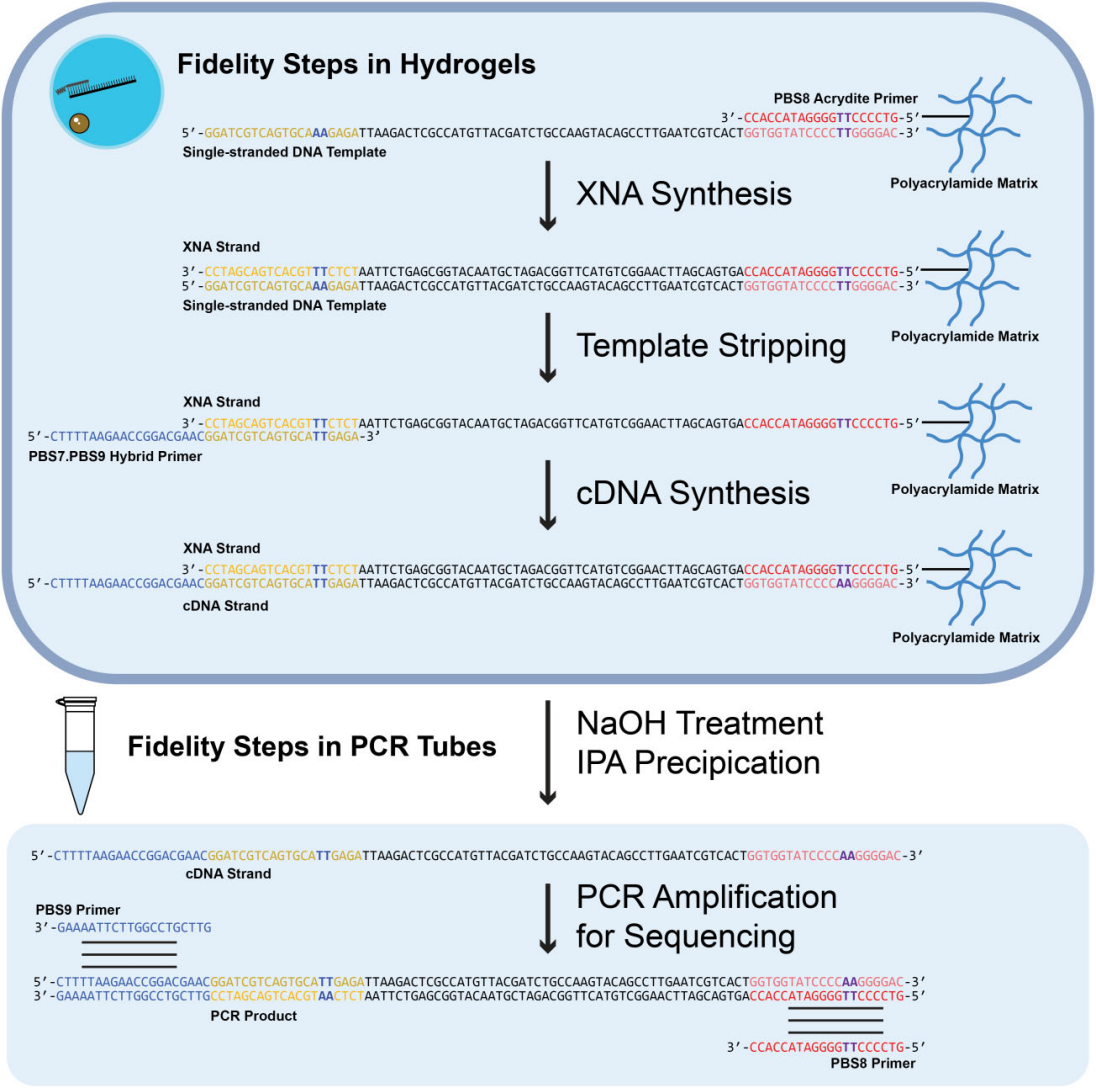
XNA fidelity assay in hydrogel particles. A DNA primer covalently linked to the hydrogel matrix is designed with a TT–TT mismatch in the primer binding site of the DNA template. The XNA strand is synthesized by primer extension using the cognate XNA polymerase. The DNA template is removed and an overhang primer designed with a second TT–TT mismatch is hybridized to the newly synthesized XNA strand. The XNA strand is reverse transcribed with Bst DNA polymerase. cDNA is isolated using isopropanol precipitation, amplified, cloned, and sequenced. Sequences containing both TT–AA watermarks are used to measure fidelity.

Next, we devised a strategy to unambiguously demonstrate that cDNA molecules isolated from an XNA replication cycle were the products of XNA transcription and reverse transcription events rather than a by-product of PCR (Fig. 2). To eliminate any possibility of contamination by the starting DNA template, the upstream and downstream primer binding sites (PBS) of the starting DNA template were designed to contain dinucleotide mismatches that would be corrected following a complete replication cycle. Accordingly, the 5′ PBS contained an AA dinucleotide that would be converted to TT, and the 3′ PBS contained a TT dinucleotide that would be converted to AA. The presence of these transitions in the sequenced product was viewed as watermarks to ensure that replication passed through the XNA intermediate. Additional controls included a 5′ unpaired region in the RT primer that was designed to introduce a unique PBS for PCR and a no RT control reaction intended to identify DNA contaminants that might arise by PCR (Supplementary Figs S2 and S3).

The hydrogel-coated magnetic particles used for this study were prepared by bulk mixing. Accordingly, paramagnetic beads were combined with a 6% acrylamide–bisacrylamide mixture, a 5′-modified acrydite DNA primer, and an ammonium persulfate solution. An oil mixture containing the TEMED catalyst was added to the top of the acrylamide mixture, forming a biphasic solution. Polymerization of the hydrogel coat was initiated by emulsification of the biphasic solution, producing monodispersed hydrogel particles (Supplementary Fig. S1A). Once the beads were washed to remove the excess oil, the primer-functionalized hydrogel particles were manually counted with a hemacytometer, and the concentration was calculated for use in the fidelity assay.

XNA transcription was performed by annealing the DNA template to the DNA primer cross-linked in the hydrogel matrix. Upon addition of the DNA template, the hydrogel particles were briefly heated at 95°C for 3 min and cooled on ice to promote formation of the primer–template duplex. The DNA template was copied into XNA by supplying the XNA polymerase and cognate xNTPs to the hydrogel particles and incubating the reaction mixture at 55°C in 1× ThermoPol buffer [20 mM Tris–HCl, 10 mM (NH_4_)_2_SO_4_, 10 mM KCl, 2 mM MgSO_4_, 0.1% Triton X-100, pH 8.8]. Following XNA synthesis, the DNA template was removed upon denaturation with NaOH buffer at 37°C to promote duplex destabilization. The hydrogel particles were neutralized with breaking buffer [10 mM Tris–HCl, pH 7.5, 100 mM NaCl, 1 mM EDTA, 1% (v/v) SDS, and 1% (v/v) Triton X-100] and washed repeatedly to remove the reagents from the XNA transcription reaction.

The XNA strand covalently attached to the hydrogel matrix was reverse transcribed following a similar primer extension procedure. Following hybridization of the DNA primer, the hydrogel particles were incubated with Bst DNA polymerase and dNTPs at 50°C to promote cDNA synthesis. Bst DNA polymerase was chosen based on its known ability to copy a range of XNA templates into DNA [39]. The cDNA was removed with NaOH buffer at 37°C, recovered, neutralized with 3 M NaOAc buffer, and precipitated with isopropanol. The resulting pellet was washed with cold 70% ethanol, dried, resuspended in nuclease-free water, amplified, cloned, and sequenced. Only sequences containing the watermarks were used to calculate the aggregate fidelity of replication.

**Figure 3.**
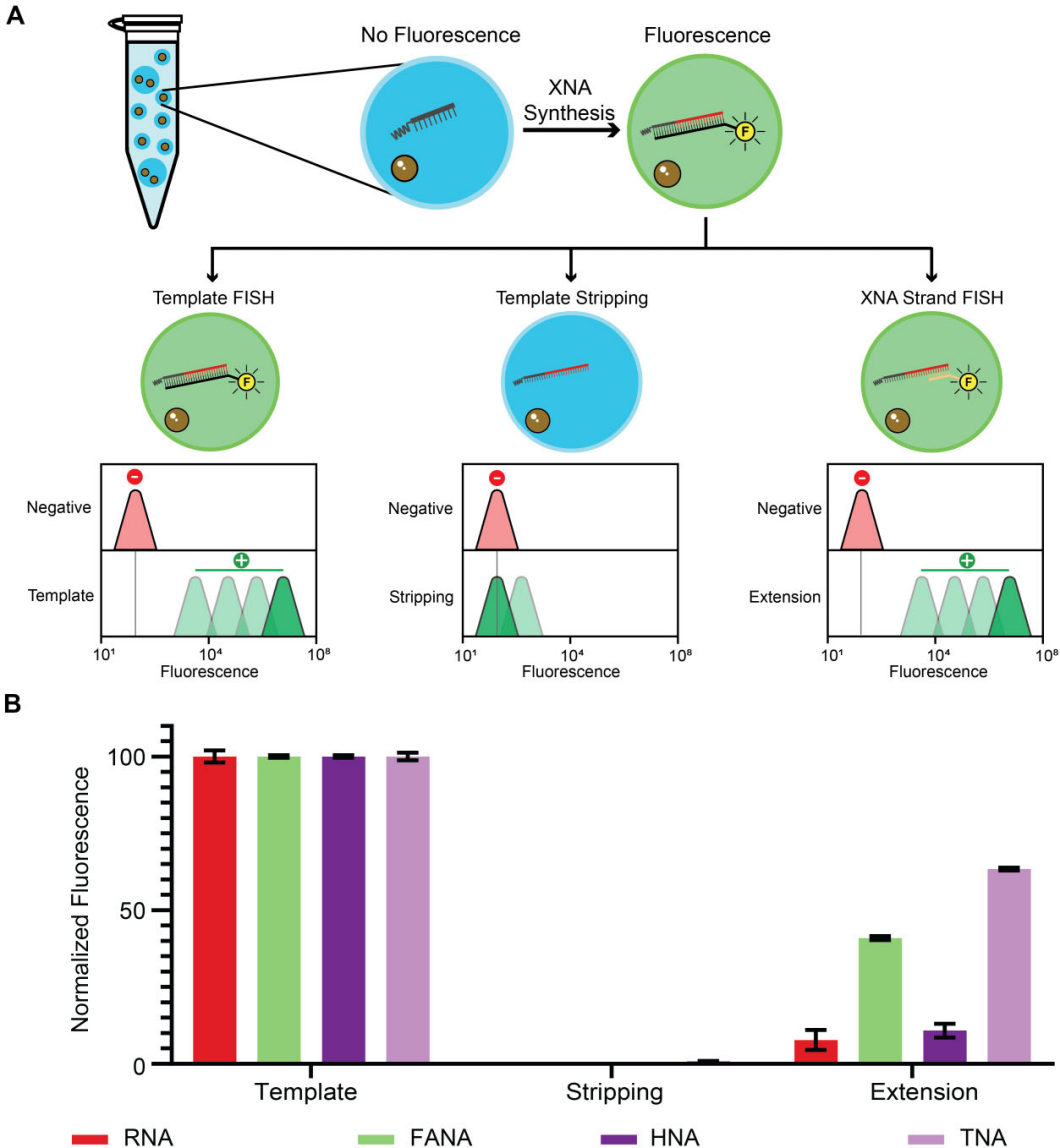
Evaluating XNA synthesis by FISH. (**A**) Schematic depiction of the FISH assay performed on hydrogel particles with expected flow cytometry values. (**B**) Fluorescence values observed for XNA synthesis in hydrogel particles after XNA synthesis, template stripping, and FISH probing (SEM, *n* = 3).

To evaluate the efficiency of XNA synthesis, a FISH assay was used to assess primer occupancy and full-length product formation by flow cytometry (Fig. 3). Accordingly, DNA primers cross-linked to the hydrogel matrix were annealed to 5′-FAM-labeled DNA template and extended with XNA. Flow cytometry analysis shows high fluorescence shifts across all XNA systems. This signal disappears when the template is removed, indicating that the DNA template is no longer hybridized to the XNA strand or present within the hydrogel matrix. We confirmed the presence of full-length XNA product by probing the 3′ end of the XNA strand (2′ end for TNA) with a 5′-FAM-labeled DNA probe. Flow cytometry measurements reveal that ∼60% of the signal remains for TNA, ∼40% for FANA, and ∼10% for HNA and RNA. We attribute the lower signal levels observed for HNA and RNA to their difficulty of synthesis and stability in the hydrogel matrix, respectively. In the case of RNA, it is also possible that the template stripping conditions with NaOH may have led to RNA degradation, as Tgo-QGLK is known to have strong RNA synthesis activity [32].

**Figure 4.**
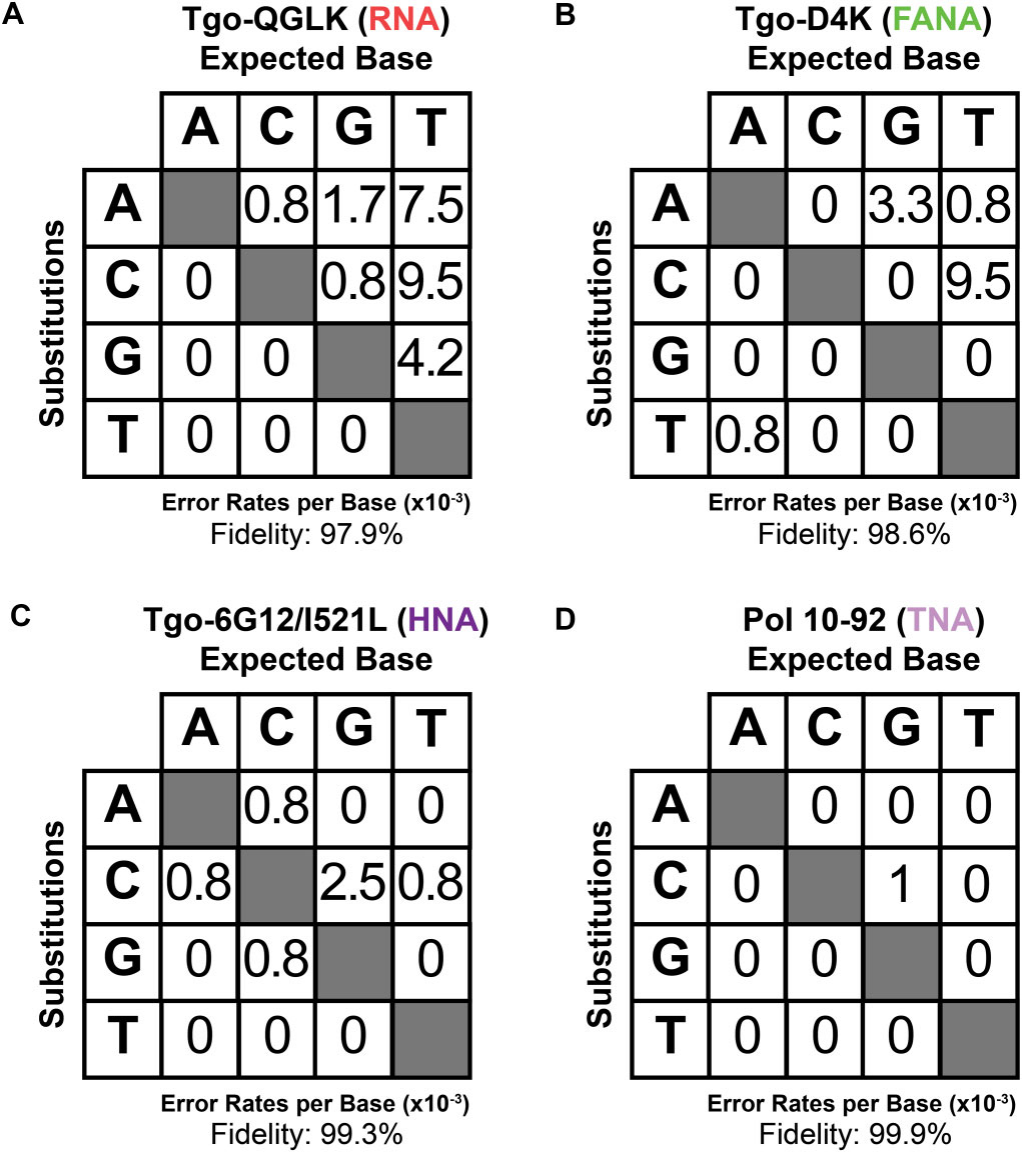
XNA polymerase fidelity. Mutational profile observed for (**A**) RNA synthesis using Tgo-QGLK, (**B**) FANA synthesis using Tgo-D4K, (**C**) HNA synthesis using Tgo-6G12/I521L, and (**D**) TNA synthesis using pol 10–92. In each case, Bst DNA polymerase was used to copy the XNA strand back into DNA. Fidelity data derive from sequencing ≥1000 nucleotide positions. Values were not corrected for random mistakes made during reverse transcription or PCR.

Having confirmed that full-length XNA product is observed in all cases, we proceeded to evaluate the fidelity of replication using the hydrogel strategy. XNA polymerase fidelity was calculated using an aggregate fidelity score, involving the sequencing of >1000 nucleotide incorporation events from a full XNA replication cycle (DNA → XNA → DNA). This strategy provides a more complete view of the replication cycle than single nucleotide kinetic measurements, identifying INDELs and mutations that occurred during XNA synthesis (DNA → XNA) and XNA reverse transcription (XNA → DNA). After aligning sequences, aggregate fidelity was calculated based on the number of mutations, insertions, and deletions observed in the sequences. The engineered RNA polymerase exhibited an aggregate fidelity score of 97.9% (Fig. 4A and Supplementary Fig. S4); although no aggregate fidelity score has been reported, an error rate of 0.0013% was previously determined by sequencing a synthesized 1.7 kb messenger RNA encoding luciferase by the RNA polymerase [32]. The difference in reported fidelities may be due to template sequence bias or differences in estimating error rates by considering reverse transcriptase and Taq DNA polymerase error. Both the HNA and FANA polymerases exhibited aggregate fidelity scores that closely match their previously reported scores of 99.7% and 98.6%, respectively (Fig. 4B and C, and Supplementary Figs S5 and S6) [9]. The TNA polymerase (pol 10–92), the latest TNA synthesizing enzyme developed to date [33], exhibited an aggregate fidelity score of 99.9% (Fig. 4D and Supplementary Fig. S7).

The hydrogel particle display format described in this method has several advantages relative to more traditional bead-based assays. First, instead of attaching the DNA primer directly to the surface of a bead, the primer is distributed throughout the matrix of a polyacrylamide gel. This step allows for greater scaling of the particles by avoiding the need to chemically derivatize the bead surface and oligonucleotide prior to conjugation. For example, derivatizing beads with DNA by click chemistry, a standard approach for DNA-encoded library strategies, requires modifying the bead surface with a suitable alkyne and converting the amino group on a 5′-amine-modified DNA primer into an azide [40]. The hydrogel strategy streamlines the particle conjugation process by eliminating these steps from the protocol. It also provides a more favorable solution-like environment for enzymatic reactions, which may help with reproducibility. Additionally, hydrogel particles are easier to evaluate by flow cytometry than conventional beads, which are prone to aggregation and more difficult to flow as individual particles. At this time, template length limitations have not been explored with the hydrogel particle format and may be a factor to consider when choosing templates. However, potential constraints on template length may be ameliorated with larger hydrogel shells to avoid steric hinderance. As described by Paegel and co-workers, the size of the hydrogel shells can be increased simply choosing magnetic beads with larger diameters (>2.5 μm) [27].

Although the method is written as an approach for measuring aggregate fidelity values by taking an XNA system through a cycle of replication, the method could be adapted to study only the reverse transcription step. In this case, the XNA template itself would be prepared by solid-phase synthesis with a 5′ acrydite group and hydrogel particle formation would proceed by replacing the 5′ acrydite primer with the newly synthesized 5′ acrydite XNA strand. Then, following hydrogel particle formation and validation by flow cytometry, the reverse transcription step would be evaluated by copying the XNA strand into DNA, amplifying the DNA by PCR, and cloning and sequencing the resulting cDNA product.

In summary, we have shown that hydrogel particle display offers a versatile and cost-effective method for measuring the fidelity of engineered polymerases that are currently being developed for applications in synthetic biology and biomedicine. In contrast to the traditional bulk solution approach, the hydrogel strategy offers a purification-free strategy that can be completed in 1–2 days on scales that are 10–30-fold lower than previous approaches. The lower reaction scale translates to significant cost savings of valuable xNTPs, many of which are expensive and/or require custom laboratory synthesis. Taken together, these benefits suggest that polymerase fidelity could soon become a routine part of the polymerase discovery pipeline rather than a final characterization step once the engineering or evolutionary steps are complete. By incorporating this information into the discovery process, it may be possible to develop enzymes that are superior to those that have been developed to date, as fidelity is a key parameter of polymerase function. Overall, we suggest that the benefits of increased speed and reduced consumables make hydrogel particles an ideal format for assessing XNA polymerase fidelity.

## Supplementary Material

gkaf038_Supplemental_File

## Data Availability

All data are available upon reasonable request.

## Supplementary data

Supplementary data is available at NAR online.

## References

[1] Aschenbrenner J, Marx A. DNA polymerases and biotechnological applications. Curr Opin Biotechnol. 2017; 48:187–95. 10.1016/j.copbio.2017.04.005. 28618333

[2] Loeb LA, Monnat RJ Jr DNA polymerases and human disease. Nat Rev Genet. 2008; 9:594–604. 10.1038/nrg2345. 18626473

[3] Wu WJ, Yang W, Tsai MD. How DNA polymerases catalyse replication and repair with contrasting fidelity. Nat Rev Chem. 2017; 1:0068 10.1038/s41570-017-0068.

[4] Tindall KR, Kunkel TA. Fidelity of DNA synthesis by the *Thermus aquaticus* DNA polymerase. Biochemistry. 1988; 27:6008–13. 10.1021/bi00416a027. 2847780

[5] Goodman MF, Creighton S, Bloom LB et al. Biochemical basis of DNA replication fidelity. Crit Rev Biochem Mol Biol. 1993; 28:83–126. 10.3109/10409239309086792. 8485987

[6] Creighton S, Bloom LB, Goodman MF. Gel fidelity assay measuring nucleotide misinsertion, exonucleolytic proofreading, and lesion bypass efficiencies. Methods Enzymol. 1995; 262:232–56. 10.1016/0076-6879(95)62021-4. 8594351

[7] McInerney P, Adams P, Hadi MZ. Error rate comparison during polymerase chain reaction by DNA polymerase. Mol Biol Int. 2014; 2014:287430 10.1155/2014/287430. 25197572 PMC4150459

[8] Nikoomanzar A, Chim N, Yik EJ et al. Engineering polymerases for applications in synthetic biology. Q Rev Biophys. 2020; 53:e8 10.1017/S0033583520000050. 32715992

[9] Pinheiro VB, Taylor AI, Cozens C et al. Synthetic genetic polymers capable of heredity and evolution. Science. 2012; 336:341–44. 10.1126/science.1217622. 22517858 PMC3362463

[10] Nikoomanzar A, Vallejo D, Yik EJ et al. Programmed allelic mutagenesis of a DNA polymerase with single amino acid resolution. ACS Synth Biol. 2020; 9:1873–81. 10.1021/acssynbio.0c00236. 32531152

[11] Hoshino H, Kasahara Y, Kuwahara M et al. DNA polymerase variants with high processivity and accuracy for encoding and decoding locked nucleic acid sequences. J Am Chem Soc. 2020; 142:21530–37. 10.1021/jacs.0c10902. 33306372

[12] Liu C, Cozens C, Jaziri F et al. Phosphonomethyl oligonucleotides as backbone-modified artificial genetic polymers. J Am Chem Soc. 2018; 140:6690–99. 10.1021/jacs.8b03447. 29722977

[13] Lelyveld VS, Fang Z, Szostak JW. Trivalent rare earth metal cofactors confer rapid NP-DNA polymerase activity. Science. 2023; 382:423–29. 10.1126/science.adh5339. 37883544 PMC10886449

[14] Chen T, Hongdilokkul N, Liu Z et al. Evolution of thermophilic DNA polymerases for the recognition and amplification of C2′-modified DNA. Nat Chem. 2016; 8:556–62. 10.1038/nchem.2493. 27219699 PMC4880425

[15] Siegmund V, Santner T, Micura R et al. Screening mutant libraries of T7 RNA polymerase for candidates with increased acceptance of 2′ modified nucleotides. Chem Commun. 2012; 48:9870–72. 10.1039/c2cc35028a. 22932771

[16] Freund N, Taylor AI, Arangundy-Franklin S et al. A two-residue nascent-strand steric gate controls synthesis of 2′-*O*-methyl- and 2′-*O*-(2-methoxyethyl)-RNA. Nat Chem. 2023; 15:91–100. 10.1038/s41557-022-01050-8. 36229679 PMC7614059

[17] Culbertson MC, Temburnikar KW, Sau SP et al. Evaluating TNA stability under simulated physiological conditions. Bioorg Med Chem Lett. 2016; 26:2418–21. 10.1016/j.bmcl.2016.03.118. 27080186

[18] Koshkin AA, Nielsen PE, Meldgaard M et al. LNA (locked nucleic acid): an RNA mimic forming exceedingly stable LNA:LNA duplexes. J Am Chem Soc. 1998; 120:13252–53. 10.1021/ja9822862.

[19] Nielsen PE, Egholm M, Berg RH et al. Sequence-selective recognition of DNA by strand displacement with a thymine-substituted polyamide. Science. 1991; 254:1497–500. 10.1126/science.1962210. 1962210

[20] Wilds CJ, Damha MJ. 2′-Deoxy-2′-fluoro-beta-D-arabinonucleosides and oligonucleotides (2′F-ANA): synthesis and physicochemical studies. Nucleic Acids Res. 2000; 28:3625–35. 10.1093/nar/28.18.3625. 10982885 PMC110742

[21] Lee EM, Setterholm NA, Hajjar M et al. Stability and mechanism of threose nucleic acid toward acid-mediated degradation. Nucleic Acids Res. 2023; 51:9542–51. 10.1093/nar/gkad716. 37650628 PMC10570051

[22] Watts JK, Katolik A, Viladoms J et al. Studies on the hydrolytic stability of 2′-fluoroarabinonucleic acid (2′F-ANA). Org Biomol Chem. 2009; 7:1904–10. 10.1039/b900443b. 19590787

[23] Wang Y, Ngor AK, Nikoomanzar A et al. Evolution of a general RNA-cleaving FANA enzyme. Nat Commun. 2018; 9:5067 10.1038/s41467-018-07611-1. 30498223 PMC6265334

[24] Sau SP, Fahmi NE, Liao J-Y et al. A scalable synthesis of α-L-threose nucleic acid monomers. J Org Chem. 2016; 81:2302–7. 10.1021/acs.joc.5b02768. 26895480

[25] Liao J-Y, Bala S, Ngor AK et al. P(V) reagents for the scalable synthesis of natural and modified nucleoside triphosphates. J Am Chem Soc. 2019; 141:13286–89. 10.1021/jacs.9b04728. 31298849

[26] Nikoomanzar A, Dunn MR, Chaput JC. Engineered polymerases with altered substrate specificity: expression and purification. Curr Protoc Nucleic Acid Chem. 2017; 69:4.75.1–20. 10.1002/cpnc.33. 28628207

[27] Cavett V, Chan AI, Cunningham CN et al. Hydrogel-encapsulated beads enable proximity-driven encoded library synthesis and screening. ACS Cent Sci. 2023; 9:1603–1610. 10.1021/acscentsci.3c00316. 37637732 PMC10451030

[28] Yik EJ, Medina E, Paegel BM et al. Highly parallelized screening of functionally enhanced XNA aptamers in uniform hydrogel particles. ACS Synth Biol. 2023; 12:2127–34. 10.1021/acssynbio.3c00189. 37410977

[29] Lozoya-Colinas A, Yu Y, Chaput JC. Functionally enhanced XNA aptamers discovered by parallelized library screening. J Am Chem Soc. 2023; 145:25789–96. 37962593 10.1021/jacs.3c09497 PMC10690791

[30] Houlihan G, Arangundy-Franklin S, Holliger P. Engineering and application of polymerases for synthetic genetics. Curr Opin Biotechnol. 2017; 48:168–79. 10.1016/j.copbio.2017.04.004. 28601700

[31] Cavicchioli R. Archaea—timeline of the third domain. Nat Rev Microbiol. 2011; 9:51–61. 10.1038/nrmicro2482. 21132019

[32] Cozens C, Pinheiro VB, Vaisman A et al. A short adaptive path from DNA to RNA polymerases. Proc Natl Acad Sci USA. 2012; 109:8067–72. 10.1073/pnas.1120964109. 22566643 PMC3361454

[33] Maola VA, Yik EJ, Hajjar M et al. Directed evolution of a highly efficient TNA polymerase achieved by homologous recombination. Nat Catal. 2024; 7:1173–85. 10.1038/s41929-024-01233-1.

[34] Yu H, Zhang S, Chaput JC. Darwinian evolution of an alternative genetic system provides support for TNA as an RNA progenitor. Nat Chem. 2012; 4:183–87. 10.1038/nchem.1241. 22354431

[35] Wang Y, Wang Y, Song D et al. A threose nucleic acid enzyme with RNA ligase activity. J Am Chem Soc. 2021; 143:8154–63. 10.1021/jacs.1c02895. 34028252

[36] Eremeeva E, Fikatas A, Margamuljana L et al. Highly stable hexitol based XNA aptamers targeting the vascular endothelial growth factor. Nucleic Acids Res. 2019; 47:4927–39. 10.1093/nar/gkz252. 30968117 PMC6547419

[37] Rose KM, Alves Ferreira-Bravo I, Li M et al. Selection of 2′-deoxy-2′-fluoroarabino nucleic acid (FANA) aptamers that bind HIV-1 integrase with picomolar affinity. ACS Chem Biol. 2019; 14:2166–75. 31560515 10.1021/acschembio.9b00237 PMC7005942

[38] Taylor AI, Pinheiro VB, Smola MJ et al. Catalysts from synthetic genetic polymers. Nature. 2015; 518:427–30. 10.1038/nature13982. 25470036 PMC4336857

[39] Jackson LN, Chim N, Shi C et al. Crystal structures of a natural DNA polymerase that functions as an XNA reverse transcriptase. Nucleic Acids Res. 2019; 47:6973–83. 10.1093/nar/gkz513. 31170294 PMC6649750

[40] Cochrane WG, Malone ML, Dang VQ et al. Activity-based DNA-encoded library screening. ACS Comb Sci. 2019; 21:425–35. 10.1021/acscombsci.9b00037. 30884226 PMC6786493

